# Hepatocellular Carcinoma Prevention in the Era of Hepatitis C Elimination

**DOI:** 10.3390/ijms241814404

**Published:** 2023-09-21

**Authors:** Jeffrey V. Lazarus, Camila A. Picchio, Massimo Colombo

**Affiliations:** 1 HPAM, CUNY Graduate School of Public Health and Health Policy (CUNY SPH), New York, NY 10027, USA; jeffrey.lazarus@isglobal.org; 2 Faculty of Medicine and Health Sciences, University of Barcelona, 08036 Barcelona, Spain; 3 Barcelona Institute for Global Health (ISGlobal), Hospital Clínic, University of Barcelona, 08036 Barcelona, Spain; camila.picchio@isglobal.org; 4 EASL International Liver Foundation, 1203 Geneva, Switzerland

**Keywords:** hepatitis C virus, direct-acting antiviral therapy, sustained virological response, hepatocellular carcinoma, surveillance, ultrasound, alpha-fetoprotein, liver stiffness

## Abstract

The hepatitis C virus (HCV), a single-stranded RNA virus belonging to the Flaviviridae family, is a major cause of hepatocellular carcinoma (HCC) worldwide. Tumors caused by HCC have an increased mortality rate globally, which is more accentuated in Western countries. The carcinogenic potential of this virus is mediated through a wide range of mechanisms, spanning from the induction of chronic inflammation to oxidative stress and deregulation of cellular pathways by viral proteins. As the number of new infections continues unabated, HCC-related mortality should be prioritized through early detection, continued prevention of HCV transmission, and treatment of HCV with safe and efficacious direct antiviral agents (DAAs). People who inject drugs (PWID) are a significant reservoir of new HCV infections globally, and in order to eliminate hepatitis C as a global health threat, as set out by the World Health Organization, an integrated approach based on the optimization of care delivery and increased access to harm reduction and treatment for PWID is needed. Thanks to the development of safe and effective antiviral agents, eradication of the infection is now possible in almost all treated patients, leading to a significant reduction but not the elimination of the risk for HCC in cured patients. This is particularly relevant among aged populations who have cofactors of morbidity known to accelerate HCC progression, such as diabetes, obesity, and excessive alcohol consumption. Given the restless accumulation of individuals with cured HCV infection, the implementation of risk-stratified surveillance programs becomes impellent from a cost-effectiveness perspective, whereas the availability of a performant biomarker to predict HCC in cured patients remains an unmet clinical need.

## 1. Introduction

The hepatitis C virus (HCV) is a major etiologic agent of hepatocellular carcinoma (HCC) around the world. The expression of its oncogenic potential is modulated by environmental, lifestyle, and genetic factors [1]. There are an estimated 58.5 million people living with HCV globally that are responsible for 1.75 million new infections every year and 187,000 incident cases of liver cancer causing some 150,000 related deaths. Overall, more than 350,000 deaths are related to hepatitis C every year [2,3]. In 2020, 20% of liver cancer cases were due to chronic HCV and more than 50% of liver cancers are attributable to HCV in countries with the highest burdens, such as the United States, Pakistan, and Egypt. According to the Global Burden of Disease study 2019, HCV accounted for 45% of cases of incident HCC deaths in females and 22% of incident HCC deaths in men globally, i.e., HCV was the first and second leading cause of liver cancer deaths for females and males, respectively [4]. Globally, the incidence of HCC is increasing, with recent data predicting an increase from 841,000 cases in 2018 to 1.4 million cases in 2040 and increasing trends of mortality rates in countries with a low Human Development Index score [5]. In 2022, the World Health Organization (WHO) updated the Global Health Sector Strategy (GHSS) on Viral Hepatitis 2016–2021, which includes the goal of reducing the incidence and mortality of viral hepatitis by 2030 [6].

Despite the availability of efficient, curative, and safe direct-acting antiviral (DAA) therapy to treat hepatitis C, the new HCV infections reported annually surpass the number of people being treated. This gap results in an increased number of people living with HCV at lifelong risk of developing liver complications, including HCC [3]. As only one-fifth of all viremic individuals have been identified, currently, the targets set out by the WHO to eliminate HCV as a global health threat by 2030 are not likely to be reached. Furthermore, half of incident cases occur among high-risk groups, including people who inject drugs (PWID), and therefore the elimination of hepatitis C requires an integrated approach based on optimization of care delivery and increased access to harm reduction services, which include combining opioid agonist treatment and needle/syringe provision to reduce exposure to communicable agents [3]. Reaching the cases of HCV which remain undiagnosed will require unique models of care that tailor to the needs of at-risk populations and use innovative strategies to conduct this [7]. To reinvigorate the political will in the elimination of HCV, the European Association for the Study of the Liver (EASL) has suggested the positioning of hepatitis C in the context of Europe’s efforts to prevent cancer [8]. Along this line, the International Liver Cancer Association (ILCA) has recently addressed regulatory authorities in a white paper which recapitulates the interventions needed to reduce mortality from HCC. Apart from the prevention of risk factors, special attention should be given to the recognition of liver disease in the individual person, with an emphasis on the recognition of who among individuals with liver disease is at risk of developing HCC. This allows for the surveillance of those who are at significant risk of developing a tumor and to provide treatment at a stage where a cure is still possible [9]. Despite treatment of chronic HCV infection and reaching a sustained virological response (SVR), the risk of HCC and HCC-related mortality is substantially reduced but not eliminated [10,11,12,13,14]. The residual risk of HCC among DAA-treated patients with HCV followed for longer than three years was much higher in patients with cirrhosis than in those with METAVIR stage 3 fibrosis (1.8 per 100 person-years (95% CI, 0.88–3.80) vs. 0.6 per 100 person-years (95% CI, 0.3–1.2) [15,16]. his may have relevant clinical consequences as guideline-recommended semi-annual screening is no longer cost-effective with the approximately halved annual incidence rate (≤1%) in patients who achieved an HCV cure [14]. Such a wide heterogeneity of HCC incidence among hepatitis C patients who have achieved SVR emphasizes the importance of risk stratification algorithms for cost-effective screening programs that aim to increase early diagnosis and the curing of liver cancer.

## 2. Hepatitis C Elimination, where Do We Stand?

In 2016, the WHO called for the elimination of viral hepatitis as a public health threat by 2030 [6]. HCV elimination targets were defined in the WHO Global Health Sector Strategy (GHSS) 2016–2021 and data were updated in the GHSS 2022–2030 [17]. Compared to 2020, and in line with the strategy, the HCV incidence should decline from 1.57 million to 350,000 in 2030, and the HCV mortality target is to drop from 290,000 deaths in 2020 to 140,000 in 2030. In order to reach these targets, increasing screening, diagnosis, and linkage to care is essential and, therefore, HCV diagnosis should increase from 30% of all patients diagnosed in 2020 to 90% in 2030. Of these, 80% should be receiving treatment by the year 2030 in comparison to 30% in 2020 [17]. A study assessing the progress towards HCV elimination in high-income countries between 2017 and 2019 concluded that of the 45 included countries, only 11 were on track to meet the WHO’s elimination targets by 2030 (Australia, Canada, France, Germany, Iceland, Italy, Japan, Spain, Sweden, Switzerland, and United Kingdom), and 27 countries were not expected to achieve elimination before 2050 [18] (Table 1). The COVID-19 pandemic has slowed down progress on HCV elimination, which can in turn impact HCC incidence. A modeling study estimated that between 2020 and 2030, 72,300 excess liver-related deaths will occur as a consequence of COVID-19 pandemic-related interruptions of HCV services in the year 2020 [19]. One promising approach, that has been taken up globally, is the micro-elimination of HCV [20]. Through a focus on populations with a high prevalence of HCV, for example, those on dialysis or co-infected with HIV, HCV can be progressively eliminated, which will subsequently impact HCC incidences. By adhering to the micro-elimination strategy, it is possible to improve cure outcomes among those treated.

## 3. Mechanisms of Liver Cell Carcinogenesis by HCV

The pathogenesis of HCV-related HCC is a multifactorial process where the crucial mechanism is persisting liver cell inflammation, as documented by the clear association that exists between HCC and cirrhosis. In certain clinical contexts, however, liver inflammation has been shown to translate into a favorable histologic biomarker of HCC outcome, largely depending on the ability that cell infiltrates retain to circumvent immunotolerance and dispose of transformed hepatocytes [21]. The inflammatory stimuli resulting from persistent replication of HCV are elicited by the virus, being able to evade the virus’ neutralizing response of the host immunity, thus allowing HCV to hijack the homeostatic mechanisms of the liver cells, an event that in parallel stimulates the restless deposition of fibrotic tissue and promotes the neoplastic transformation of the hepatic parenchyma [21,22,23].

The pillar of HCV-induced liver inflammation is an immune cell-mediated attack on the infected liver cells, which accounts for the release of reactive oxygen species (ROS) and pro-inflammatory cytokines by both liver and immune cells, including natural killer cells and T cells [21]. On their own, the resulting inflammation and necrosis of the liver cells act as a potent stimulus to hepatocyte regeneration and wound healing, with significant consequences on the process of oxidative stress in the liver, which leads to the induction of epigenetic and oncogenic alterations, telomere shortening, and, in the end, to genomic instability [23]. The process of fibrotic remodeling of the liver has important prognostic implications as it partners with the process of liver carcinogenesis driven by specific viral proteins like core proteins and the non-structural NS5A protein that are able to subvert liver cell homeostasis [24,25,26,27]. The core proteins are involved in the dysregulation of lifesaving functions of the liver cell such as growth, differentiation, apoptosis, transcription, and angiogenesis. The bridge between those dysregulations and HCV is the activation of the MAPK, Wnt/beta-catenin, TGF-alfa, PI3K/Akt/mTOR, NF-kB, IL-6/STAT3, and androgen receptor signaling, whereas the protective apoptotic signaling becomes suppressed. Partnering with the oncogenic activity of the core protein of HCV is the NS5A protein that engages with such relevant pro-oncogenic pathways as beta-catenin, PI3K/AKT/mTOR, NF-kB, and p53. The final consequences of all those interactions are the remodeling of the chromatin structure, a shelter for the nuclear DNA, coupled with a reshuffle of cell gene expressions leading to altered epigenetic regulation and the production of microRNAs [28].

These epigenetic events include the increase in DNA methyltransferase activity and histone deacetylation, whereas the HCV-induced increase in the expression of the pro-oncogenic microRNA miR-155 leads to activation of the Wnt signaling implicated in the accelerated proliferation and neoplastic transformation of the liver cells [29,30]. Other important steps in HCV-induced liver carcinogenesis are the onset of endoplasmic reticulum stress and the interaction with the gut microbiota. At the endoplasmic reticulum level, HCV infection causes an accumulation of misfolded proteins, which activates the unfolded protein response and the release of calcium ions into the cytoplasm. Calcium is released in the cytoplasm and may stimulate ROS production that can induce inflammation, tissue damage, and fibrosis and contribute to the development of HCC [23].

Another cytoplasmic event that may contribute to HCC onset is steatosis, i.e., the accumulation of triglycerides in hepatocytes due to HCV’s core ability to reduce triglyceride transfer protein activity and cause oxidative stress that contributes to the oncogenic process [31]. The altered composition of the gut microbiota has been involved in HCV-related HCC following studies with whole-genome sequencing of fecal DNA from patients with HCV-related HCC and the demonstration that the transplantation of microbiota from patients with HCC into mice amplified liver cancer incidence as compared with mice with transplanted microbiota from healthy donors [32].

## 4. Recognition of People Living with HCV at Risk of HCC

Severe liver disease, metabolic co-morbidities, and lifestyle factors including alcohol, tobacco, and limited physical activity are associated with an increased risk of HCC in patients with chronic HCV infection [15]. Patients with cirrhosis and METAVIR F3 liver fibrosis due to chronic HCV infection are at risk of developing HCC at a rate greater than the 1.5% per year threshold. Based on this threshold, EASL recommends surveillance with an abdominal ultrasound given that it is a cost-effective strategy [15,33]. Given that HCV eradication by antiviral treatment reduces but does not eliminate HCC risk, these same recommendations hold true for patients with a similar stage of disease who have been treated and cured for chronic HCV infection.

In a landmark study in Veterans Affairs (VA) hospitals, USA, the annual risk of HCC in DAA-cured patients with advanced hepatitis C was 1.8%, much lower than that in untreated patients or patients who failed to respond to HCV therapy (2.8%) [12]. Of note, HCC risk in patients achieving an HCV cure correlated with the coexistence of other morbidities like diabetes, overweight/obesity, smoking, and heavy alcohol use, with combinations of risk factors being often synergistic rather than additive [34,35,36]. Between 2013 and 2020, more than 10 million people with chronic HCV infection initiated DAA therapy, one-third of them being in Egypt, leading to an accumulation of people who developed HCC after achieving SVR. In a hepatitis C disease burden simulation model (HEP-SIM), which aimed to simulate the population of people in the United States with HCV who would be considered candidates for HCC surveillance, HCC incidence was predicted to fall from 30,000 people in 2012 to 13,000 in 2040, whereas the number of SVR candidates for screening was predicted to rise from 1000 to 6000 per 100,000/year.

Similarly, the percentage of candidates who have achieved SVR and who would need to undergo HCC surveillance was estimated to rise from 8.5% in 2012 to 64.6% in 2040 [12]. Thus, the exponential growth of HCV-cured individuals who need HCC surveillance has fostered the race towards cost-effective screening programs grounded on risk stratification, mainly focused on the identification of patients with advanced liver disease. The more widely adopted strategy has been the use of the FIB-4 score, a simplified scoring system that assesses liver disease severity by combining the routine chemistries transaminases and platelet count with patient age. According to EASL and the American Association of the Study of the Liver (AASLD) recommendations, a cut-off > 3.25 identifies patients who likely have cirrhosis, whereas in a retrospective review of the VA cohort of patients with cured HCV, the same cut-off of FIB-4 was able to separate non-cirrhotic patients with a >2% annual risk of HCC from similar patients with a <0.5% annual risk of malignant transformation of the liver [37,38,39,40].

Non-invasive assessment of liver stiffness with elastography techniques like Fibroscan^®^ and acoustic radiation force impulse (ARFI) have gained popularity in identifying advanced liver disease both in patients with current HCV infection and those with cured hepatitis C. While these procedures were not specifically developed to assess HCC risk, evaluation of liver stiffness is generally recommended as a secondline approach after patient stratification for liver disease severity using inexpensive routine tests like FIB-4 or the aspartate aminotransferase to platelet ratio index (APRI) [37,38]. EASL recommends surveillance of patients with METAVIR stage F3 fibrosis diagnosed with Fibroscan^®^ at a cut-off of 10–13 kPa and with ARFI at a cut-off of 1.6 and 2.17 m/s. Non-invasive identification of patients with cirrhosis can be achieved either with serum or transient elastography such as a Fibroscan^®^ at a cut-off of > 12.5 kPa, ARFI > 2.17 m/s, FIB-4 > 3.25, or APRI > 2.

Recent attempts to reinforce clinical algorithms for HCC prediction through the incorporation of polygenic scores were met with little success. In a prospective study of cirrhotic patients with cured HCV or alcoholic disease, a model incorporating six single-nucleotide polymorphisms (SNIPs) for the lipid metabolism and one SNIP for Wnt-beta-catenin into an aMAP score built on age, sex, albumin, bilirubin, and platelet count failed to predict HCC with respect to aMAP alone [41]. Likewise, genetics do not seem to help in screening the general population for other cancers. In a recent modeling study from the UK, screening for prostate, breast, and colon cancer showed no survival benefits when the standard screening tests were coupled with a polygenic score compared to the standard screening tests alone [42]. Noticeably, risk-stratified screening might be used with a collateral scope, like to provide less intensive screening to low-risk individuals in order to reduce the unnecessary harms and costs of over-screening, tailoring screening age range, frequency, and method to each risk group. Not surprisingly, the implementation of a risk-stratified surveillance program for HCC is scattered with multiple barriers, which span from the histological and molecular heterogeneity of the tumor to the lack of external validation and calibration of the current biomarkers, except for alpha-fetoprotein (AFP), and the time dependence of the outcomes.

## 5. Recommended Strategies of HCC Surveillance

Secondary prevention based on semi-annual surveillance is associated with improvements in early tumor detection and reduced HCC mortality [9,15]. Although it is highly operator-dependent and has worse performance in patients with obesity, ultrasound is the standard of care imaging modality recommended for HCC surveillance by all liver societies [15,43,44] (Table 2). EASL recommends semi-annual abdominal ultrasound exams, without determination of serum AFP level, not only for HCV patients with cirrhosis but also for those with the METAVIR F3 stage of fibrosis. The same holds true for patients with cured HCV infection and a similar disease stage [15]. AASLD recommends against surveillance of patients with advanced fibrosis but without cirrhosis [43]. Though insufficient as a standalone biomarker for HCC screening, AFP has a role in conjunction with other tests for the early detection of HCC [15,43,44].

Along these lines, ILCA recommends delivering surveillance with ultrasound not only to those with cirrhosis but also to those with METAVIR stage F3 fibrosis and high scores of GALAD, a phase III validated biomarker that includes gender, age, AFP-L3, AFP, and des-gamma—carboxy prothrombin (DCP) level [10,45,46]. However, further translational studies are required before GALAD is endorsed as the ideal biomarker for risk-stratified surveillance of HCV patients. Bi-annual examination with abdominal ultrasounds is widely recognized to confer significant clinical benefits in virtue of its ability to identify liver cancer at a curable stage. In a meta-analysis and systemic review of 59 studies comparing 41,052 patients with an HCC detected by surveillance and 104,344 patients with an incident HCC, surveillance was associated with an odds ratio gain of 1.86 (95% CI 1.73–1.98) in terms of early-stage detection, 1.83 (95% CI 1.69–1.97) in terms of receipt of curative therapy, and 0.67 (95% CI 0.61–0.72) in terms of reduced mortality. Interestingly, all those clinical benefits came at the expense of mild-severity harms that affected 8.8–27.8% of the individuals [33]. In a previous meta-analysis of 32 studies with 13,367 patients, the same group reported ultrasound alone to have a satisfactory specificity of 91% (95% CI 86–94%) for T1/T2 HCC but quite a low diagnostic sensitivity of 47% (95% CI 33–61%) only, that however could be inflated to 63% (95% CI 48–75%) with the combined determination of serum AFP level [47]. Though associated with superior survival compared to annual surveillance (40.3 vs. 30 months, *p* = 0.03), semi-annual surveillance is not further improved with quarterly surveillance [48,49]. Noticeably, the recommendations of the international societies are not fully aligned with each other, and they show nuances with respect to the use of liver biopsy and second-level imaging techniques to achieve the final diagnosis of HCC (Table 2).

Surveillance is not recommended in patients with clinical decompensation when liver transplantation is not an option. However, a large grey area between these two recommendations is represented by aged patients with comorbidities, where the lack of data prevents the adoption of any specific recommendation and decisions are taken on individualized bases. In one modeling study among HCV-cured patients with advanced fibrosis, bi-annual HCC surveillance with ultrasound and AFP was considered cost-effective up to the age of 60, because it added 23 quality-adjusted life years and detected 24 potentially curable HCCs per 1000 patients [50]. This fuels the debate with respect to the growing population of aged patients with a cured hepatitis C, where HCV eradication is associated with a reduced (not abolished) risk of HCC coupled with an increase in longevity due to a significant reduction in mortality from liver decompensation and extrahepatic complications of HCV [51].

## 6. Overcoming the Underuse and Low Diagnostic Accuracy of Currently Available Screening Tests

Apart from the inadequate risk stratification of the patients, working against the effectiveness of HCC surveillance are several other hurdles that include the underuse of surveillance and the suboptimal accuracy of currently available screening tests. To overcome the underuse of screening, one intervention aiming to increase patients’ compliance with screening was the development of programs of mail outreach coupled with specific training of nurses and dedicated pathways to screening that have proven to be of some efficacy [52]. Two-phase CT and contrast-enhanced MRI can yield superior sensitivity for early-stage HCC detection compared to ultrasound (86% vs. 29%, respectively), but their use as surveillance tests is limited by concerns about cost, radiologic capacity, and potential adverse effects by contrast and/or radiation exposure [53,54].

Abbreviated magnetic resonance imaging (AMRI) is a user-friendly approach that allows to overcome the low sensitivity of ultrasound for early tumor detection and its suboptimal specificity, leading to screening-related harms. AMRI might better serve certain subgroups of patients, including obese individuals, patients with nonalcoholic steatohepatitis, and those with Child–Pugh B or C cirrhosis. In a meta-analysis of 15 studies involving more than 2800 patients (917 with HCC), AMRI showed a sensitivity of 86% for any size tumor and of 69% for tumors < 2 cm in size [55]. In that systemic review, the sensitivity of ultrasound was definitively lower than that of AMRI, whereas the specificity (94%) of non-contrast AMRI was comparable to that of contrast-enhanced AMRI. While the great appeal of non-contrast AMRI is built on low invasiveness, low cost, and repeatability, all those pros are counterbalanced by technical constraints like the lower Contrast-to-Noise Ratio (CNR) and dependence on Diffusion-Weighted Imaging (DWI) that may challenge the identification of HCC nodules [56,57]. In a recent analysis of privately insured patients, those with cirrhosis were likely to incur both out-of-pocket and opportunity costs from HCC screening [58]. This was particularly true for patients undergoing second-level imaging techniques and, in general, for low-risk patients, in whom potential harms related to increases in overdiagnosis offset the minimal benefits of screening.

## 7. The Future Prospects

**Figure 1:**
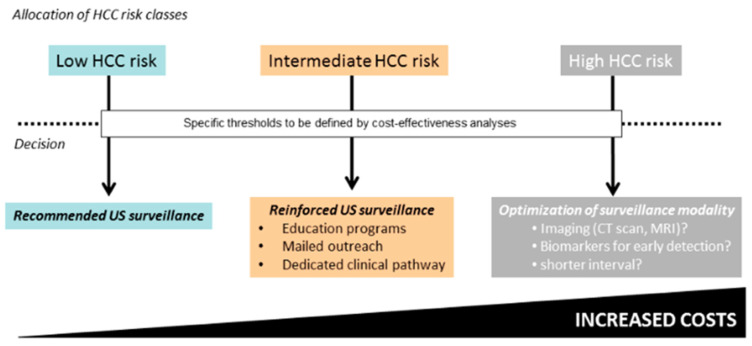
Potential application of HCC risk stratification using scoring systems [61]. Abbreviations: hepatocellular carcinoma (HCC); magnetic resonance imaging (MRI); United States (US).

In countries where hepatitis C elimination campaigns are running with success, the population that has achieved SVR and would be considered candidates for HCC surveillance is expanding. This expansion is occurring at a rate that will level off or outpace the canonical population with HCV-related HCC, thereby emphasizing the need for cost-effective strategies of HCC surveillance [14,18]. The lack of performant biomarkers for risk-stratified screening of individuals at risk of developing HCC is becoming a major barrier to the optimization of secondary prevention of this lethal disease. At present, there are a number of phase II studies aimed at validating a heterogeneous array of experimental blood-based biomarkers for the implementation of programs of early diagnosis of HCC. More promising biomarkers were vehicles of small RNA clusters like the extracellular vesicles; cell-free DNA combined with gender, age, AFP, and desgamma carboxy prothrombin(DCP); and methylation markers of 28 genes combined with AFP, AFP-L3, DCP, age, and ex, all demonstrating at least 90% specificity and from 74% to 100% sensitivity [59]. Once prospectively and externally validated, performant biomarkers might allow for risk-stratified surveillance to become a standard of care in the setting of liver cancer that would affect the secondary prophylaxis of HCV-related HCC, too [60] (Figure 1). A simplified algorithm of surveillance was recently proposed based on bi-annual ultrasound for low-risk patients, whereas patients at high risk of HCC should be more aggressively screened in terms of imaging modality with AMRI or CT scan and intensified screening intervals [61].

In the grey area between the two wings of HCC risk are those patients with intermediate risk in whom surveillance should be reinforced through education programs, mailed outreach, and dedicated pathways. Such a risk-stratified surveillance program is currently challenged by the lack of validated cut-offs for risk stratification, not to speak about the concern of granting increased access to second-level imaging techniques like AMRI or CT scans for millions of screening candidates worldwide. As the future of surveillance of infectious diseases is going to be shaped by emerging forms of technology driven by artificial intelligence [61], a way forward to monitor HCV and its complications could be the integrated utilization of biosensors, quantum computing, augmented intelligence, and language models like chat-GPT that might help in the process of early warning, pathogen classification, risk assessment, source identification, hotspot detection, tracking, and forecasting. An example of the application of artificial intelligence in the educational activity was the questionnaire-based study conducted in two liver transplant centers in the US that investigated the power of chat-GPT to empower patients and improve health literacy in the cirrhosis and HCC domains. The survey highlighted certain limitations of chat-GPT, which, in fact, reported better responses on basic knowledge, lifestyle, and treatment than those regarding diagnosis and preventing medicine, providing comprehensive responses to 41.1% of the 164 questions graded by only two transplant hepatologists [62]. Last, but not least, a push to eliminate hepatitis C as a form of primary prevention of HCC could be provided by a protective vaccine, owing to the fact that despite outstanding progress being made in pharmacotherapy of HCV, new infections continue to outpace achieving SVR. Unfortunately, despite promising interactions with virus replication, to date, vaccines have failed to be protective [63].

## Figures and Tables

**Table 1:** Seven years after WHO member states committed to eliminate viral hepatitis as a public health threat by 2030 [16].

Progress in Reducing Hepatitis-Related Mortality Has been Made between 2015 and 2019
Ten-times increase in HCV treatment from 2015
98% of HBV infections and 87% of HCV infections have still not been treated
Only 10% of HBV-infected and 21% of HCV-infected persons even know their diagnosis
In Africa and South East Asia, diagnosis and treatment rates lag far behind 2030 targets
Current funding stands at less than 10% of the estimated USD 6 billion a year being required to meet hepatitis B and C elimination goals

Abbreviations: Hepatitis B virus (HBV); hepatitis C virus (HCV).

**Table 2:** Recommendations of the International Liver Societies for HCC surveillance [13,41,42].

Recommendation	EASL 2018	AASLD 2023	APASL 2017
Screening interval	Six months	Six months	Six months
Imaging	US	US, contrast	US
		enhanced MR/CT when	
		US visualization limited	
Serum AFP	No	Yes	Yes
Liver biopsy	Non-cirrhotics	Non-cirrhotics	Unsolved >/= 1 cm
		Unsolved nodules	
		LI-RADs 4 and 5 in trials	
Diagnosis of HCC	CT/MRI/CEUS	CT/MRI	CT/MRI/CEUS

Includes <1 cm nodule US every 4 months in the first year. Alternative imaging/CT/MRI every 3–6 months. If size unchanged, resume 6 months contrast medium.

## Abbreviations

**aMAP**	HCC risk calculator: age, sex, albumin, T bilirubin, platelets
**AFP**	Alpha Fetoprotein
**AFP-L3**	Alpha Fetoprotein Lecitin-3
**Akt**	serine/threonine protein kinase family member
**APRI**	Aspartate aminotransferase to platelet ratio index
**ARFI**	Acoustic Radiation Force Impulse US scan
**APASL**	Asia Pacific Association for the Study of the Liver
**AASLD**	American Association for the Study of the Liver
**Chat-GPT**	Chat Generative Pre-trained Transformer
**CT scan**	Computed tomography scan
**COVID-19**	Coronavirus Disease 2019
**CEUS**	Contrast-Enhanced Ultrasonography
**DAAs**	Direct antiviral agents
**DCP**	Des-gamma-carboxy prothrombin
**DNA**	Deoxyribonucleic acid
**EASL**	European Association for the Study of the Liver
**GALAD**	HCC risk calculator: gender × age × log alpha-fetoprotein × des-gamma-carboxy prothrombin
**GHSS**	Global Health Sector Strategy
**HBV**	Hepatitis B virus
**HCC**	Hepatocellular carcinoma
**HCV**	Hepatitis C virus
**IL-6**	Interleukin 6
**ILCA**	International Liver Cancer Association
**kPa**	kilo Pascal
**LI-RADS**	Liver Imaging Reporting and Data System
**MAPK**	Mitogen-activated protein kinase
**MRI**	Magnetic resonance imaging
**METAVIR**	Meta-analysis of histological data in viral hepatitis
**MIR-155**	MicroRNA-155
**mTOR**	Mammalian Target of Rapamycin
**NF-kB**	Nuclear factor kappa-light-chain-enhancer of activated B cells
**NS5A**	Non-Structural 5 A PI3K Phospho Inositide 3-Kinases
**PWID**	People who inject drugs
**RNA**	Ribonucleic acid
**ROS**	Reactive oxygen species
**SNIP**	Single nucleotide polymorphism
**STAT3**	Signal Transducer and Activator of Transcription 3
**SVR**	Sustained virological response
**TGF-alfa**	Tumor Growth Factor alfa
**WHO**	World Health Organization
**Wnt**	Wingless-related integration site
